# Physical Properties and pH Environment of Foam Dressing Containing *Eclipta prostrata* Leaf Extract and Gelatin

**DOI:** 10.3390/ph16050685

**Published:** 2023-05-02

**Authors:** Sukhontha Hasatsri, Jariya Suthi, Nattaporn Siriwut, Onjira Charoensappakit

**Affiliations:** 1 Department of Pharmacy Practice, College of Pharmacy, Rangsit University, Pathum Thani 12000, Thailand; 2 Sunpasitthiprasong Hospital, Ubon Ratchathani 34000, Thailand; 3 NorthEastern Institute of Child and Adolescent Mental Health, Khon Kaen 40000, Thailand; 4 Chularat Cholvaej Hospital, Chonburi 20000, Thailand

**Keywords:** *Eclipta prostrata*, gelatin, foam dressing, wound dressing, physical property, absorption, dehydration, pH environment

## Abstract

*Eclipta prostrata (E. prostrata)* has several biological activities, including antibacterial and anti-inflammatory activities, that improve wound healing. It is well known that physical properties and pH environment are crucial considerations when developing wound dressings containing medicinal plant extracts in order to create an appropriate environment for wound healing. In this study, we prepared a foam dressing containing *E. prostrata* leaf extract and gelatin. Chemical composition was verified using Fourier-transform infrared spectroscopy (FTIR) and pore structure was obtained using scanning electron microscopy (SEM). The physical properties of the dressing, including absorption and dehydration properties, were also evaluated. The chemical properties were measured to determine the pH environment after the dressing was suspended in water. The results revealed that the *E. prostrata* dressings had a pore structure with an appropriate pore size (313.25 ± 76.51 µm and 383.26 ± 64.45 µm for the *E. prostrata* A and *E. prostrata* B dressings, respectively). The *E. prostrata* B dressings showed a higher percentage of weight increase in the first hour and a faster dehydration rate in the first 4 h. Furthermore, the *E. prostrata* dressings had a slightly acidic environment (5.28 ± 0.02 and 5.38 ± 0.02 for the *E. prostrata* A and *E. prostrata* B dressings at 48 h, respectively).

## 1. Introduction

Plant-based biomaterials have several benefits over synthetic materials, including cost-effectiveness, safety for humans, and their environmentally friendly composition [1]. Developing novel wound dressings containing medicinal plant extracts can improve clinical outcomes and increase plant value [2,3]. In this study, we examined *Eclipta prostrata* L. (*E. prostrata* L.) due to its pharmacological properties. *E. prostrata* L., commonly known as False daisy, Ink plant, Bhringraj, Bhumiraj, Aali jhar, or Nash jhar, is a herbaceous plant that belongs to the family Asteraceae [4]. It is a weed that grows in moist places such as rivers, marshes, or the edge of rice fields [4,5]. It is found in many parts of the world, including Thailand, China, India, Nepal, and Brazil [4]. It has been long used in the treatment of several diseases, including coronary heart disease, diabetes, gastrointestinal diseases, respiratory diseases, skin diseases, and wounds [5]. The leaves have various biological activities, including antibacterial, antifungal, and anti-inflammatory activities, that improve wound healing [6,7,8,9]. Therefore, a wound dressing containing *E. prostrata* L. extract could be used for treating infection and inflammation in wound healing. Kang et al. [10] suggest that *E. prostrata* L. extract is a potential treatment for inflammatory skin conditions such as atopic dermatitis. The *E. prostrata* L. extract improved the allergic inflammation of the skin by restoring the skin barrier dysfunction, decreasing epidermis/dermis thickness, and regulating the immune balance [10]. Raoul et al. evaluated wound healing in rats after applying an ointment containing *E. prostrata* leaf extract [11]. The results showed that the wounds treated with medicinal ointment completely healed faster than when treated with Vaseline^®^ and Cicatryl^®^ [11]. Babu et al. [12] developed a hydrogel containing *E. prostrata* leaf extract and evaluated its physical properties, including pH, viscosity, and spreadability. However, no studies have yet developed a wound dressing containing *E. prostrata* leaf extract in sheet form or as foam dressings. Wound dressing selection is based on the wound’s cause, location, healing phase, exudate level, pain, odor, infection, size, and depth [13]. For deep or tunneling wounds, packing wounds with dressing in the form of rope is essential for promoting wound healing from the inside [14]. On the other hand, superficial or partial-thickness wounds require dressing in the form of a sheet to cover the wound and facilitate wound function [15]. In addition, choosing the physical characteristics of wound dressings according to the exudate level will provide the optimum environment for facilitating wound healing.

The development of foam dressing for wound healing applications should focus on physical properties. According to ideal wound dressing properties, the wound dressings should be able to absorb wound exudate in order to prevent maceration (softening and breaking down of the surrounding skin due to prolonged exposure to moisture), malodor, local wound infection, and delayed wound healing [15,16,17,18,19]. However, different absorption properties of wound dressings are appropriate for different types of wounds [16,20,21]. For example, a wound dressing with a high absorption capacity should be selected for wounds with a high level of exudate. Additionally, the dehydration rate of wound dressing is also an important property that maintains a moist wound-healing environment [22]. A moist wound environment has several benefits, including increased keratinocyte migration and re-epithelialization, increased collagen synthesis, increased autolytic debridement, reduced pain, and decreased inflammation [23,24].

Elevated exudate pH correlates with an increased risk of infection and delayed wound healing [25,26]. Therefore, the ideal wound dressing should not provide an alkaline wound environment. Foam dressing’s porous structure is also an essential factor in the wound-healing process [27,28]. The wound-healing process is a complex biological process that seeks to recover damaged tissues and restore the skin’s normal function. The wound-healing process consists of four continuous and overlapping phases: hemostasis, inflammation, proliferation, and remodeling [29]. The appropriate pore size of the porous structure is essential for the proliferative phase. Both natural and synthetic polymers are used in wound dressings, and each type of polymer can help to produce a porous structure. Natural polymers are commonly chosen for wound dressing development, including chitosan, cellulose, hyaluronic acid, collagen, alginate, and gelatin [30]. Gelatin could be used to produce porous structures via the freeze-drying technique [31,32]. Porous gelatin materials support cell migration and the development of new tissue [33]. Interestingly, gelatin could also provide biodegradable and biocompatible material [31,34]. Thus, the present study aimed to develop a foam dressing containing *E. prostrata* leaf extract and gelatin; the physical properties and pH wound environment of the dressing were subsequently evaluated. The results from this study could deliver the profile of physical characteristics and pseudo-wound environment after being treated with foam dressing containing *E. prostrata* leaf extract and gelatin.

## 2. Results

### 2.1. Thickness Test

The composition of prepared foam dressings containing *E. prostrata* leaf extract and gelatin is shown in Table 1. The *E. prostrata* A dressing had a lower concentration of *E. prostrata* leaf extract than the *E. prostrata* B dressing. The *E. prostrata* dressings were soft and flexible. The *E. prostrata* A dressing had a thickness of 4.236 ± 0.0519 mm; the *E. prostrata* B dressing had a thickness of 3.945 ± 0.1403 mm. The *E. prostrata* A dressing was thicker than the *E. prostrata* B dressing.

### 2.2. Fourier-Transform Infrared Spectroscopy (FTIR)

**Figure 1:**
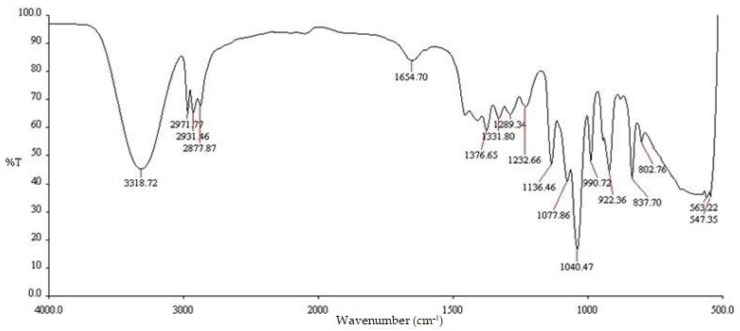
FTIR spectra of the *Eclipta prostrata (E. prostrata)* leaf extract.

**Figure 2:**
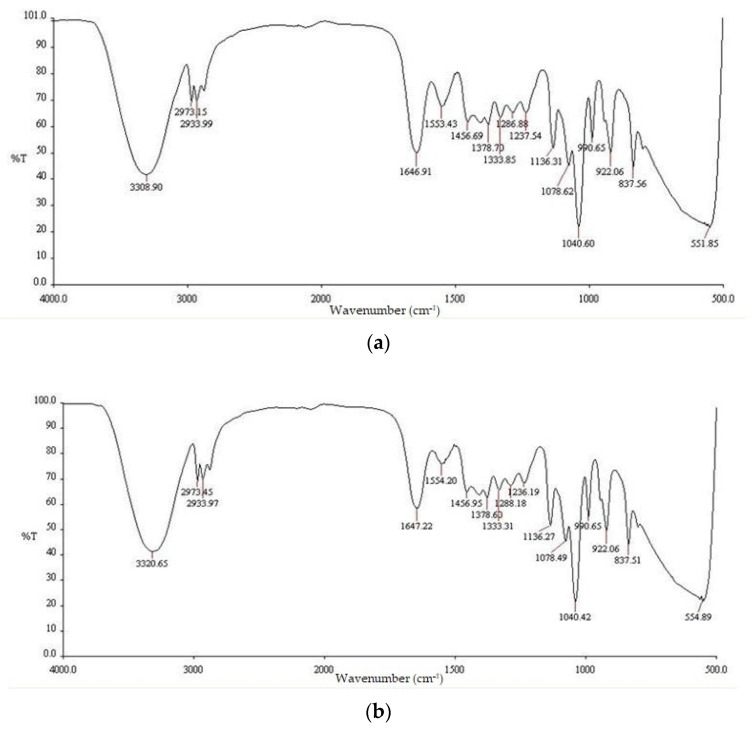
FTIR spectra of the (**a**) *E. prostrata* A dressing and (**b**) *E. prostrata* B dressing.

Table 1 shows the FTIR peak values and functional groups of the *E. prostrata* leaf extract and the *E. prostrata* A and B dressings. Figure 1 shows the FTIR spectra of the *E. prostrata* leaf extract. Figure 2 shows the FTIR spectra of the *E. prostrata* A and B dressings. After mixing gelatin and *E. prostrata* leaf extract and then lyophilization or freeze drying, there was an increased intensity in the functional groups (as seen in Figure 2), including amide I (1646.91 cm^−1^ and 1647.22 cm^−1^ for the *E. prostrata* A and B dressings, respectively), and amide II (1553.43 cm^−1^ and 1554.20 cm^−1^ for the *E. prostrata* A and B dressings, respectively). The amide I (mainly related to the C=O stretching vibration) and II (mainly related to the N-H bending vibration and the C-N stretching vibration) bands are associated with the presence of gelatin [35,36].

### 2.3. Morphological Properties

**Figure 3:**
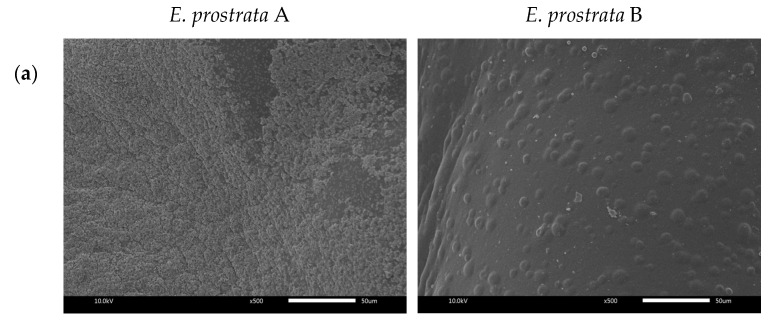

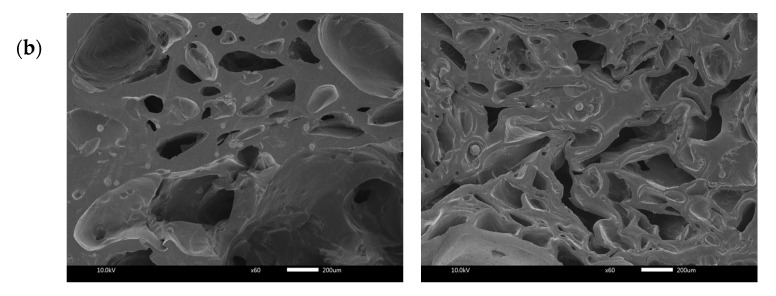
Morphological observation of (**a**) surface and (**b**) cross-section of *E. prostrata* dressings by SEM.

The surface and cross-sectional morphologies of *E. prostrata* dressings were observed using SEM (Figure 3). The size, shape, and distribution of pores are shown in Figure 3b. The average pore sizes were 313.25 ± 76.51 µm and 383.26 ± 64.45 µm for the *E. prostrata* A and B dressings, respectively (Figure 3b). The *E. prostrata* B dressing had more consistent porosity than *E. prostrata* A dressing. 

### 2.4. Absorption Properties

**Figure 4:**
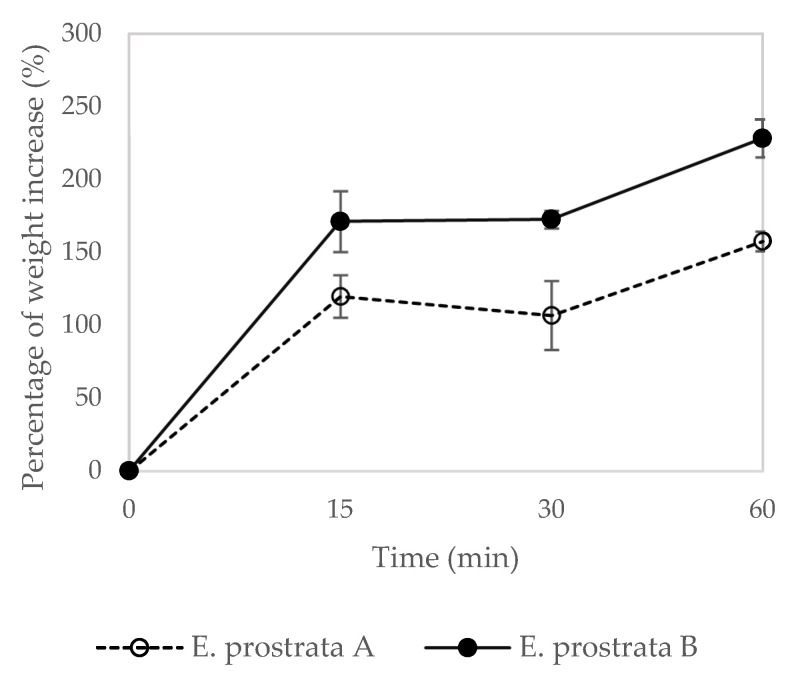
Absorption properties of *E. prostrata* A and B dressings.

The absorption properties of the dressing determine how well it can manage wound exudate and promote wound healing. The percentage of weight increase of the *E. prostrata* dressing for different periods is shown in Figure 4. The *E. prostrata* A dressing showed a lower percentage of weight increase than the *E. prostrata* B dressing. The higher *E. prostrata* leaf extract promotes the higher absorption capacity.

### 2.5. Dehydration Properties

**Figure 5:**
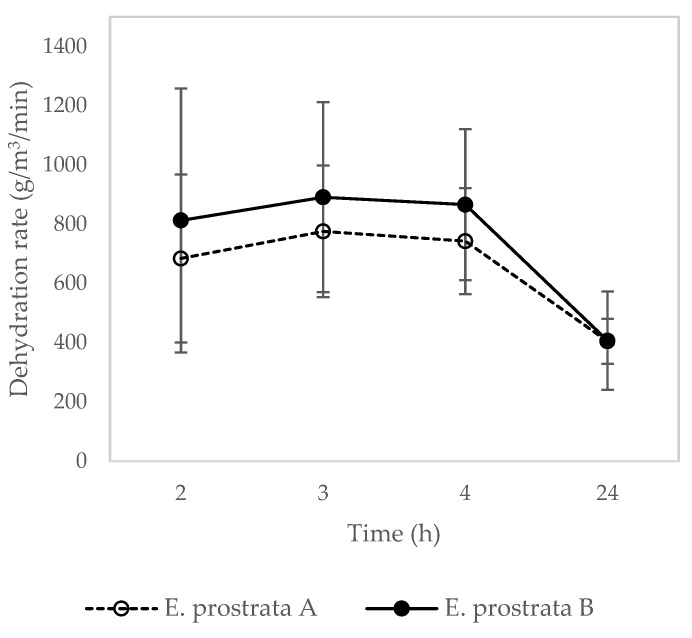
Dehydration properties of *E. prostrata* A and B dressings.

The dehydration rate of the *E. prostrata* dressing for different periods is presented in Figure 5. The *E. prostrata* B dressing showed a higher dehydration rate in the first 4 h than the *E. prostrata* A dressing. The higher *E. prostrata* leaf extract promotes the higher dehydration rate.

### 2.6. pH Measurement

**Figure 6:**
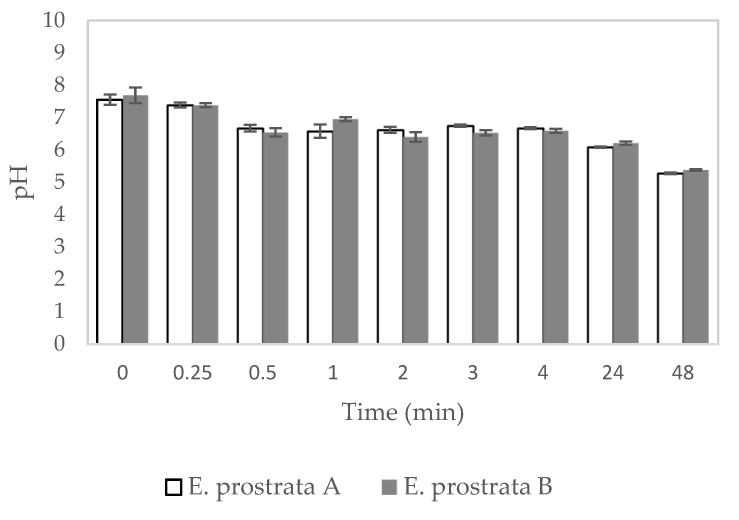
The pH of deionized water with the submerged *E. prostrata* A and B dressings.

Figure 6 demonstrates the pH of deionized water after the *E. prostrata* dressings were submerged in it. The pH of the deionized water with the submerged *E. prostrata* A dressing decreased from 7.55 ± 0.16 to 5.28 ± 0.02. In the same way, the pH of deionized water with the submerged *E. prostrata* B dressing decreased from 7.69 ± 0.24 to 5.38 ± 0.02.

### 2.7. Dispersion Characteristics

**Figure 7:**
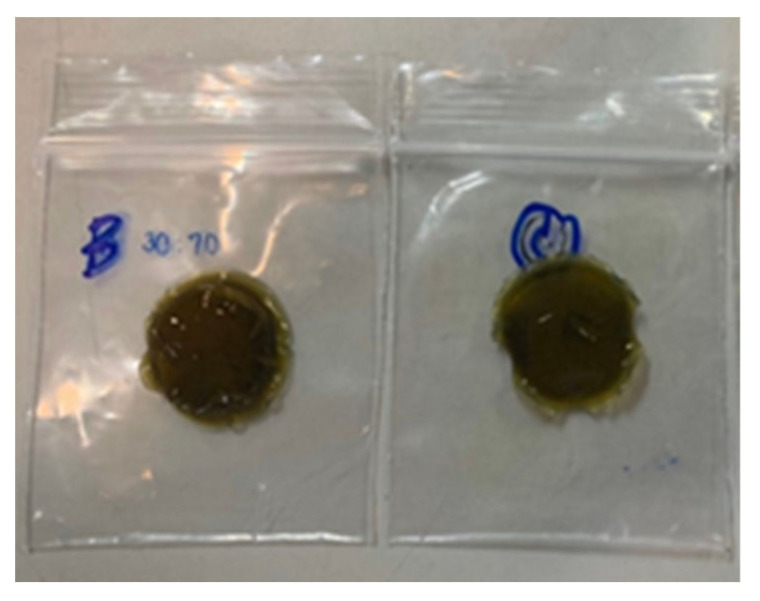
Dispersion Characteristics of (**left**) *E. prostrata* A and (**right**) *E. prostrata* B dressings.

**Figure 8:**
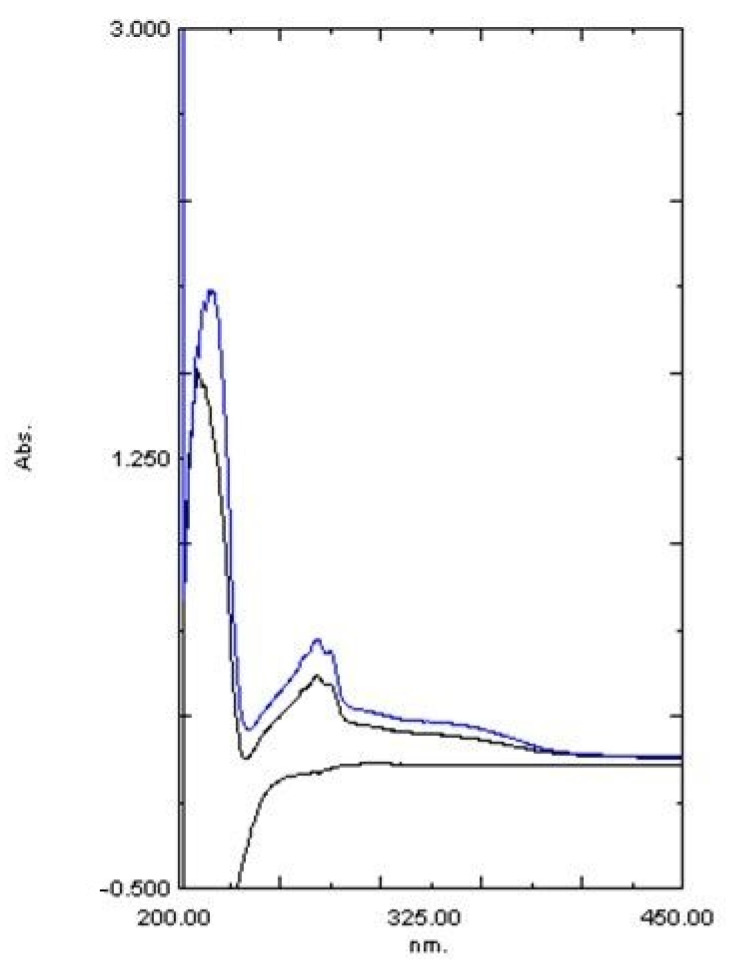
Dispersion Characteristics compared with a pseudo-wound exudate. Spectrum of (**bottom line in black**) NaCl/CaCl_2_·H_2_O solution, (**blue line**) NaCl/CaCl_2_·H_2_O solution after with the *E. prostrata* A dressing were submerged, and (**upper line in black**) NaCl/CaCl_2_·H_2_O solution after with the *E. prostrata* B dressing were submerged.

The dispersion characteristics of the *E. prostrata* dressings are shown in Figure 7 and Figure 8. The *E. prostrata* A and B dressings did not change much from their original structure after they were immersed in pseudo-wound exudate for 60 s at 100 revolutions per minute (Figure 7). However, the spectra of the pseudo-wound exudate after the *E. prostrata* A and B dressings were submerged in it were not similar to those of the pseudo-wound exudate (Figure 8).

## 3. Discussion

*E. prostrata* leaf extract has been studied for its potential wound-healing benefits, including its antimicrobial and anti-inflammatory properties [6,7,8,9]. Prior studies examining the development of wound healing products containing *E. prostrata* focus only on ointment and hydrogel formulations [11,12]. No studies have developed wound dressings containing *E. prostrata* leaf extract in sheet form or foam dressings. Developing wound dressing in sheet form has several advantages, including preventing trauma, minimizing external contamination, absorbing exudate, and keeping a wound in an optimally moist environment [16].

In this study, a foam dressing containing *E. prostrata* extract and gelatin was developed to evaluate the physical properties and pH wound environment. The *E. prostrata* dressings were soft and flexible. These properties help to maintain a moist wound environment, reduce the risk of maceration, and allow use in the movement areas, such as the knee or elbow [37]. The *E. prostrata* A dressing was thicker than the *E. prostrata* B dressing. This is explained as the high protein content in bovine gelatin increased the polymer matrix’s solids content. Hence, the increase in gelatin or protein concentration has induced an increase in the thickness of the foam dressing [38]. However, the thickness was unrelated to the absorption and dehydration properties, as shown in Figure 4 and Figure 5.

The FTIR spectra are used to identify the functional groups present in the *E. prostrata* dressing, as compared to the *E. prostrata* leaf extract. It was found that the FTIR spectra of *E. prostrata* dressing had an increased intensity in the functional groups, including amide I and II (Figure 2). The amide I and II bands in the FTIR spectra are commonly used to identify the presence of gelatin [35,36]. The amide I band in FTIR spectra is a strong absorption peak that corresponds to the stretching vibration of the C=O bond in the peptide backbone [39]. This band of gelatin appears in the region of 1600–1700 cm^−1^ [35]. As shown in Figure 2, the amide I band was around 1646–1648 cm^−1^, indicating the presence of a predominantly random coil structure [39]. The amide II band in the FTIR spectra I also provide information on the vibrational bands of the protein backbone [39]. This band corresponds to the bending vibration of the N-H bond (40–60% of the potential energy) and the stretching vibration of the C-N bond (18–40%) in the protein backbone [39]. In the case of gelatin, the amide II band appears in the region of 1565–1520 cm^−1^ [35]. As shown in Figure 2, the amide II band was around 1553–1555 cm^−1^. The amide II band is often used in combination with the amide I band to confirm the presence of gelatin. Therefore, it indicates that our process to develop an *E. prostrata* dressing did not affect the structural property of gelatin. Gelatin could provide a porous structure and produce biodegradable and biocompatible material [31,34].

The resulting SEM image provides information about the morphology or porous structure of the *E. prostrata* dressing (Figure 3). A porous structure is crucial in wound healing because it allows cell migration and proliferation [28,40]. When a wound occurs, the first phase of wound healing is hemostasis, with vascular constriction, platelet aggregation, degranulation, and fibrin clot formation [29]. Hemostasis helps to stop bleeding, and inflammatory cells (neutrophils, monocytes, macrophages, and lymphocytes) migrate into the wound, triggering the inflammatory response (also known as the “inflammatory phase”) [29]. The next phase is proliferation, with re-epithelialization, angiogenesis, collagen synthesis, and extracellular matrix (ECM) formation; this generally overlaps with the inflammatory phase [29]. The porous structure supports this phase. Fibroblasts and endothelial cells need to migrate into the wound bed in order to proliferate and form granulation tissue at the site of injury [29]. A porous structure allows for these cells to migrate into the wound bed, promoting efficient wound healing. Following cell proliferation, the final phase is remodeling (collagen remodeling and vascular maturation) and regression [29]. A previous study by Murphy et al. [41] showed that a mean pore size of 325 µm facilitated the highest cell attachment and proliferation when compared with pores in the 85–190 µm range. As seen in Figure 3b, our SEM images of the cross-section show an average pore size of around 300 µm. This ensured that the *E. prostrata* A and B dressings had an appropriate pore size for efficient wound healing. Nevertheless, the *E. prostrata* B dressing had a more consistent porosity than the *E. prostrata* A dressing. The effect of this difference in porosity between the *E. prostrata* A and B dressings could result in differences in absorption ability.

We developed the *E. prostrata* dressing, which was designed with a porous structure, in order to increase absorption ability. In this study, absorption ability was obtained by using pseudo-wound exudate. The *E. prostrata* B dressing exhibited a stronger absorption ability than the *E. prostrata* A dressing (Figure 4). The absorption ability of the *E. prostrata* B dressing was derived from a higher-density porous structure (Figure 3b). The ideal wound dressing must absorb excess wound exudate and provide a moist environment [18,21,22,42]. Wound exudate or wound drainage is the fluid that discharges from a wound during the healing process [43]. The mechanism of exudate formation is usually due to inflammation or infection [43]. The amount of exudate produced can vary depending on the type and severity of the wound. A moist wound environment is necessary for the wound-healing process to occur effectively. An optimal moisture level enhances cell migration and proliferation, reduces pain and discomfort, and reduces infection rates [22,23]. Macerated peri-wound skin can lead to an increased risk of infection, whereas desiccated peri-wound skin can lead to decreased epithelial migration and cell death [22,44]. Therefore, the selection of absorbent wound dressing depends on the amount of exudate to prevent both maceration and desiccation. Moreover, a moist environment promotes autolysis or breakdown of necrotic tissue, called autolytic debridement [23,45]. In our previous work [20], the commercial hydrocolloid dressing and hydrocolloid with foam layer dressing had the lowest absorption capacity. Therefore it is an appropriate dressing for wounds with a low amount of exudate. In this study, both the *E. prostrata* A and B dressings had absorption characteristics similar to commercial hydrocolloid dressings and hydrocolloid with foam layer dressings [20]. These absorption characteristics meant that both the *E. prostrata* A and B dressings could be chosen for wounds with low exudate.

Dehydration rate is also essential to control the moisture balance of the wound and enhance wound healing as a result of water-retaining properties [22,46]. This can be achieved through the use of appropriate wound dressings that are designed to manage moisture levels and prevent dehydration. In addition, the selection of wound dressing also depends on the amount of exudate produced by the wound. The *E. prostrata* B dressing showed a higher dehydration rate than the *E. prostrata* A dressing. This can be explained by the higher-density porous structure of the *E. prostrata* B dressing (Figure 3b). Therefore, the *E. prostrata* B dressing can dehydrate exudate to rapidly create a moist wound-healing environment.

The pH of the wound environment is an essential factor for wound healing. The pH of healthy human skin is in the range of 5.4 to 5.9, which is slightly acidic. [47]. Propionibacterium is commonly found on human skin. Propionibacterium grow well at pH 6.00–6.50 [48]. *Staphylococcus aureus* is a pyogenic bacterium [49]. *S. aureus* prefers a neutral pH environment for optimal growth and survival [50]. Thus, an acidic environment is not favorable for harmful bacterial growth. In addition, the pH environment of chronic wounds exists at a range of 7.15 to 8.90; this is alkaline and chronic wounds are characterized by excessive protease activity [51,52,53,54,55]. Sim et al. found that faster recovery of wounded tissues was observed in wounds treated by pH 4 buffers when compared to pH 6 buffers [25]. A previous study by Leveen et al. showed that a slightly acidic environment significantly inhibits protease activity and may potentially enhance the healing of cutaneous wounds [56]. Previous studies reported fibroblast proliferation and migration behaviors associated with the acidic environment [57,58]. It means rapid wound healing occurs in an acidic environment [25,54]. We found that the *E. prostrata* A and B dressings showed similar pH decreases continuously over the period. Our *E. prostrata* dressings tended to create a slightly acidic environment. Hence, it was supposed that the *E. prostrata* A and B dressings would not interfere with the wound healing process.

The dispersion of the wound dressing refers to how well the dressing covers the wound surface. In this study, the spectra of the pseudo-wound exudate after the *E. prostrata* A and B dressings were submerged in it were not similar to those of the pseudo-wound exudate (Figure 8). In our previous study, commercial alginate dressings had the spectra of the pseudo-wound exudate after the dressings were submerged was also not similar to those of the pseudo-wound exudate [20]. Nevertheless, after interacting with the pseudo-wound exudate, the *Eclipta prostrata* A and B dressings did not change substantially from their original structure (Figure 7). It means that the *E. prostrata* dressing will not be difficult to remove. According to the spectra of the pseudo-wound exudate after the dressings were submerged, our *E. prostrata* dressings have an immediate-release formulation. The *E. prostrata* dressing should be further modified for controlled release applications by crosslinking techniques with a crosslinker, such as glutaraldehyde [59,60].

## 4. Materials and Methods

### 4.1. Materials

Gelatin (from bovine skin, gel strength 225, Type B) was purchased from Sigma–Aldrich, Inc. (St. Louis, MO, USA). *E. prostrata* leaf extract was purchased from SK Herb Co., Ltd. (Samut Sakhon, Thailand). Sodium chloride and calcium chloride dihydrate were purchased from VWR International bvba (Leuven, Belgium).

### 4.2. Preparation of Foam Dressing Containing E. prostrata Extract and Gelatin

The gelatin solution (10% *w*/*v*) was prepared by dissolving the gelatin in deionized water at 40 °C and stirring continuously for 1 h. Then, the *E. prostrata* dressing was prepared by mixing gelatin solution and *E. prostrata* leaf extract, as shown in Table 2. The mixture was stirred for 1 h to obtain a homogeneous solution. After stirring, the solution was sonicated to eliminate air bubbles and then poured into plastic plates. The plastic plates were placed into a freezer at −80 °C and frozen for 24 h. The frozen solution was then lyophilized in a freeze-dryer (SHM 021) for 48 h to become a sponge. Finally, the sponge (*E. prostrata* dressing) was slowly removed from the plastic plate. In order to prevent contamination, the *E. prostrata* dressings were then stored inside an airtight container. The composition with *E. prostrata* leaf extract of more than 40% could not prepare foam dressing.

### 4.3. Thickness Test

After lyophilization, a thickness test was performed using the Mitutoyo Dial Thickness Gauge, which provided an accuracy of 0.001 mm. Thickness was measured at five different positions (one in the center and four in the middle of each side).

### 4.4. Fourier Transform Infrared Spectroscopy (FTIR)

The *E. prostrata* leaf extracts and the *E. prostrata* dressings were recorded on a spectrum 100 FTIR Spectrometer (PerkinElmer Inc., Waltham, MA, USA) FTIR spectra were recorded from 500 to 4000 cm^−1^.

### 4.5. Morphological Properties

At a voltage of 10 kV, the *E. prostrata* dressings were examined using a Scanning Electron Microscope (SEM, JSM-IT300 JEOL). SEM with an Energy Dispersive X-ray Spectrometer (EDS) was used to analyze the dressings with the surface (500×) and cross-sectional (60×) images. The *E. prostrata* dressing was first prepared by attaching it to the aluminium stubs and then coating it with gold. This process helps to improve the conductivity of the dressing, allowing for better imaging results. The pore sizes were measured using the Image J^®^ software (National Institutes of Health, Bethesda, MA, USA).

### 4.6. Absorption Properties

The absorption properties of the *E. prostrata* dressing were examined using BS EN 13726-1: 2002, Part 1: the aspects of absorbency, Section 3.2: free swell absorptive capacities with slight modifications [61]. The *E. prostrata* dressing (2 cm × 2 cm) was prepared and weighed. A test solution (8.298 g of NaCl (0.142 mol/L) and 0.367 g of CaCl_2_2H_2_O (0.0025 mol/L)) was added to one liter of deionized water, representing a pseudo-wound exudate. The *E. prostrata* dressing was immersed in the test solution and then incubated at 37 °C. At different periods, the dressing was removed and weighed.

### 4.7. Dehydration Properties

The *E. prostrata* dressing (2 cm × 2 cm) was prepared and weighed. The *E. prostrata* dressing was immersed in the test solution (pseudo-wound exudate) for 30 min. Then, the dressing was removed, weighed, and incubated at 37 °C. At different periods, the dressing was weighed [62].

### 4.8. pH Measurement

The *E. prostrata* dressing (2 cm × 2 cm) was suspended in deionized water at a ratio of 1:25 (*w*/*v*). At different periods, the deionized water was measured using a pH meter (pH 700) [62].

### 4.9. Dispersion Characteristics

The dispersion characteristics of the *E. prostrata* dressing were examined using BS EN 137262: 2002, Part 1: the aspects of absorbency, Section 3.6: dispersion characteristics with slight modifications [63]. The *E. prostrata* dressing (2 cm × 2 cm) was prepared and immersed in the test solution and shaken for 60 s at 100 revolutions per minute. Then, the absorbance of the collected test solution was measured using a UV-spectrophotometer (UV-2501PC) by scanning between a wavelength of 200 and 450 nm.

### 4.10. Statistical Analysis

The experiments were performed in triplicate and represented in a mean ± standard deviation.

## 5. Conclusions

This study is the first development of wound dressing sheets containing *E. prostrata* leaf extract and gelatin. Our study investigated the physical properties and pH pseudo-wound environment of the *E. prostrata* dressing. Both the *E. prostrata* A and B dressings had an appropriate pore size (313.25 ± 76.51 µm and 383.26 ± 64.45 µm, respectively) for cell migration and proliferation in the wound healing process. Greater *E. prostrata* leaf extract produces a higher-density porous structure of foam dressing, resulting in a higher absorption capacity and faster dehydration rate. The *E. prostrata* dressings are designed for the low level of exudate due to their absorption capacity. In addition, the *E. prostrata* dressings make the environment slightly acidic. Therefore, it was supposed that our *E. prostrata* dressings would not provide favorable conditions for bacterial growth. Our results provide the wound dressing profiles that are essential for the decision of physicians to select the appropriate wound dressing according to the amount of exudate. Further experimental studies should focus on release patterns, pharmacological properties, such as antibacterial and anti-inflammatory activities, and wound healing assays.

## Figures and Tables

**Table 1:** FTIR peak values and functional groups of the *Eclipta prostrata (E. prostrata)* leaf extract and the *E. prostrata* A and B dressings.

Functional Groups	Peak Values		
	*E. prostrata* Leaf Extract	*E. prostrata* A Dressings	*E. prostrata* B Dressings
Alkane	1376.65 2877.87 2931.46 2971.77	1378.70 2933.99 2973.15	1378.60 2933.97 2973.45
Alkene	1654.70	1646.91	1647.22
Halo compound	802.76 837.70	837.56	837.51

**Table 2:** Composition of prepared foam dressings containing *E. prostrata* extract and gelatin.

*E. prostrata*	*E. prostrata* Leaf Extract: Gelatin (*v*/*v*)
A	3:7
B	2:3

## Data Availability

Data is contained within the article.
